# Study protocol for IMAGE: implementing multidisciplinary assessments for geriatric patients in an emergency department observation unit, a hybrid effectiveness/implementation study using the Consolidated Framework for Implementation Research

**DOI:** 10.1186/s43058-020-00015-7

**Published:** 2020-02-25

**Authors:** Lauren T. Southerland, Julie A. Stephens, Christopher R. Carpenter, Lorraine C. Mion, Susan D. Moffatt-Bruce, Angela Zachman, Michael Hill, Jeffrey M. Caterino

**Affiliations:** 1 Department of Emergency Medicine, The Ohio State Wexner Medical Center, 750 Prior Hall, 376 W 10th Ave, Columbus, OH 43210 USA; 2 Center for Biostatistics, Department of Biomedical Informatics, The Ohio State College of Medicine, Columbus, OH USA; 3 Department of Emergency Medicine, Washington University at St. Louis, St. Louis, MO USA; 4 College of Nursing, The Ohio State Wexner Medical Center, Columbus, OH USA; 5 Division of Thoracic Surgery, The Ohio State Wexner Medical Center, Columbus, OH USA

**Keywords:** Emergency department, Multidisciplinary, Geriatrics, CFIR, Lean six sigma, Observation unit, Functional status

## Abstract

**Background:**

Older adults in the emergency department (ED) are at high risk for functional decline, unrecognized delirium, falls, and medication interactions. Holistic assessment by a multidisciplinary team in the ED decreases these adverse outcomes and decreases admissions, but there are many barriers to incorporating this type of care during the ED visit.

**Methods:**

This is a hybrid type II effectiveness-implementation study using a pre-/post-cohort design (*n* = 380) at a tertiary care academic ED with an ED observation unit (Obs Unit). The intervention is a two-step protocol of (step 1) ED nurses screening adult patients ≥ 65 years old for geriatric needs using the Delirium Triage Screen, 4-Stage Balance Test, and the Identifying Seniors at Risk score. Patients who have geriatric needs identified by this screening but who do not meet hospital admission criteria will (step 2) be placed in the Obs Unit for multidisciplinary geriatric assessment by the hospital’s geriatric consultation team, physical therapists, occupational therapists, pharmacists, and/or case managers. Not all patients may require all elements of the multidisciplinary geriatric assessment. The Consolidated Framework for Implementation Research: Care Transitions Framework was used to identify barriers to implementation. Lean Six Sigma processes will be used to overcome these identified barriers with the goal of achieving geriatric screening rates of > 80%. Implementation success and associated factors will be reported. For the effectiveness aim, pre-/post-cohorts of adults ≥ 65 years old cared for in the Obs Unit will be followed for 90 days post-ED visit (*n* = 150 pre and 230 post). The primary outcome is the prevention of functional decline. Secondary outcomes include health-related quality of life, new geriatric syndromes identified, new services provided, and Obs Unit metrics such as length of stay and admission rates.

**Discussion:**

A protocol for implementing integrated multidisciplinary geriatric assessment into the ED setting has the potential to improve patient functional status by identifying and addressing geriatric issues and needs prior to discharge from the ED. Using validated frameworks and implementation strategies will increase our understanding of how to improve the quality of ED care for older adults in the acute care setting.

**Trial registration:**

ClinicalTrials.gov Identifier, NCT04068311, registered 28 August 2019

Contribution to the literature

- This study will add to the limited data on implementation strategies in the emergency department and short stay/observation unit settings.
- The Consolidated Framework for Implementation Research: Care Transitions Framework applies well to an observation unit, which focuses on rapid turnover and care transitions.
- An emergency department observation unit is a potential solution to the length of stay and staffing barriers to implementing multidisciplinary geriatric assessment.

## Introduction

The emergency department (ED) plays a critical role in caring for older adults with over 19 million ED visits a year, yet emergency care in the USA is not attuned to their needs [1]. During an ED visit, 76% of delirium is missed [2], 12–16% of older adults receive potentially harmful medications [3, 4], and 80% of patients presenting for a fall-related injury do not receive fall prevention counseling [5, 6]. These missed opportunities contribute to the poor outcomes seen in the 6 months after an ED visit for a fall or minor injury: 42% return to the ED, 25–35% suffer significant functional decline, and the mortality rate is 10 times higher than older adults without an ED visit [7–10]. The national multispecialty *Geriatric ED Guidelines* recognize this problem and endorse Multidisciplinary Geriatric Assessment for all high-risk ED patients [11]. Multidisciplinary assessment by geriatricians, case managers, pharmacists, and physical therapists (PTs) in the ED can identify geriatric syndromes and address needs, which leads to decreased unnecessary hospitalizations [12–18]. Studies of multidisciplinary assessment in the ED show measurable benefits with decreases in hospitalizations, intensive care unit admissions, ED revisits, and functional decline at 6 months [12, 15, 17, 19–24].

Despite these proven benefits, implementation of multidisciplinary care in EDs has been limited. Barriers to implementation include the 24/7 ED care model, cost of multidisciplinary staff [25], difficulties risk stratifying or choosing which older adults should receive the intervention [26], incentives for short length of stay in the ED and expedited care [27], and a lack of data on implementation in this dynamic and complex healthcare setting [28, 29]. The data supporting multidisciplinary geriatric assessment has come from EDs with external funding from research or philanthropic organizations. To be able to provide the benefits of multidisciplinary geriatric assessment to all patients, more information on implementation and operational models that do not rely on external funding is needed.

One operational model of multidisciplinary assessment in the ED uses an observation unit (Obs Unit) [30]. Obs Units are a promising solution to these barriers. Over 36% of EDs have Obs Units, which provide a setting for typically 8–24 more hours of further monitoring and testing [31–33]. Providing multidisciplinary assessments in an Obs Unit addresses the barriers of long stays in the ED, personnel costs, and consultant availability. For example, instead of needing to have a PT available at 8 pm when the patient is getting their ED evaluation, the patient can be kept in observation to see the PT at 8 am. This process has been shown to be feasible and effective in Obs Units around the world, but is not standard care in the USA [18, 34–37].

Currently, over 100 EDs have been accredited as geriatric EDs, and more are applying each year. However, only 10 have reached level 1 status, which requires the availability of multidisciplinary geriatric assessment in the ED setting. This study will develop a protocol incorporating risk stratification and multidisciplinary geriatric assessment. By providing clear information on ways to implement this type of program in a sustainable manner, this study has the potential to increase access to this valuable service in EDs across the nation.

This study will investigate the implementation of a two-step geriatric Obs Unit protocol which begins with (1) ED nurses using validated tools to assess the patients for fall risk, delirium, polypharmacy, and frailty. If needs are identified, and the patient does not meet admission criteria for their other medical issues, the patient is (2) placed in the Obs Unit to be evaluated by geriatricians, PTs, pharmacists, and/or case managers. A pilot of step 2 of the protocol resulted in new interventions for 76% of patients who were assessed by the multidisciplinary geriatric consultants [37]. However, we found a significant performance gap: the screening is being done in less than 2% of older patients. Nurses and physicians have received training on how to use the geriatric screening tools, and the consultant teams for multidisciplinary geriatric assessment are available, but they are rarely consulted. Monthly EHR reports show that geriatricians are consulted only five times a month, despite 1600 older adults being cared for in the Obs Unit per year. Currently, we do not know why the ED staff are choosing to assess some patients and not others, or whether the intervention would have benefitted those who did not receive it. Therefore, this study has two aims: evaluation of the implementation process in the ED and Obs Unit setting and evaluation of protocol effectiveness. Studying the implementation of this process is essential and will aid in potential dissemination of successful protocols for maximal national impact.

We will conduct a hybrid II effectiveness-implementation study with before and after cohort analysis, adapting the Consolidated Framework for Implementation Research: Care Transitions Framework (CFIR) [38] to the ED setting. We will track implementation processes and measures, and Lean Six Sigma rapid cycle process improvement will be used to overcome barriers to protocol use and fidelity. Lean Six Sigma is frequently used in our and other medical centers for process improvement and therefore will allow us to speak a common language with other centers for future dissemination. We will also report on measures needed for sustainability by continuing to monitor screening rates and consultant use while phasing out implementation support. Finally, to determine the effectiveness for patients, both operational metrics and patient-centered outcomes will be assessed using a pre-/post-cohort evaluation.

## Methods

This study is approved by its institutional review board and registered on clinicaltrials.gov (NCT04068311). The aims are to develop, implement, and sustain a two-step intervention providing ED geriatric assessments by combining (1) ED nurse-based screening for geriatric syndromes of all older ED patients with (2) multidisciplinary geriatric assessment in an Obs Unit. Secondly, it will describe the effect of this protocol on reducing functional decline after an ED visit.

### Setting

The study will be carried out in an academic, tertiary care referral ED with over 80,000 ED visits annually. Of those visits, 25.2% are made by adults ≥ 65 years old. The ED has an embedded 20 Obs Unit that is staffed 24/7 by advanced practice providers and 8 h a day by an emergency medicine physician. The ED and Obs Unit have access to 24-h social work and case management services, 18 h a day of pharmacist coverage, and 12 h a day of physical therapy coverage Monday through Saturday. The inpatient geriatric consult team prioritizes Obs Unit consult requests and is available during business hours.

External setting context includes the Joint Hospital Accreditation Council mandates on fall risk screening, several national reported quality measures (falls and ED recidivism), and the recent accreditation of geriatric ED programs through the American College of Emergency Physicians (www.acep.org/geda). The ED for this study has Level 1 Geriatric ED accreditation in part for the ability to provide and track geriatric screening, but the screening is being done inconsistently and therefore to maintain accreditation improvement in this area is required.

### Intervention

**Fig. 1:**
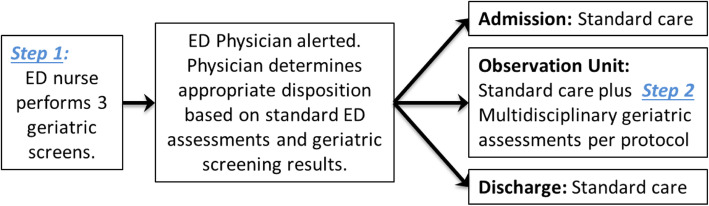
Patient flow through the ED visit and integration of the geriatric interventions per protocol

The intervention is a two-step protocol beginning with screening for geriatric syndromes for patients aged ≥ 65 years in the ED (Table 1). The screening is done by the bedside nurse and entered directly into the electronic health record (EHR). It takes 3 min, including the time needed to get the patient out of bed and to document results in the EHR [42]. Positive results are relayed to the provider team verbally or via EHR chat function. The second step is multidisciplinary assessment in the Obs Unit for those who have needs identified by the screening tools but do not meet medical necessity for this evaluation as an inpatient (Fig. 1). Patients who have acute needs and require admission or admission to a skilled nursing facility are hospitalized. For those placed in the Obs Unit, the physician team chooses which elements of multidisciplinary professionals need to be consulted based on their clinical evaluation and the geriatric screening results (Table 2).

**Table 1:** Study intervention: nurses will perform three geriatric screening assessments that direct the need for geriatrician, pharmacist, PT, and case manager evaluations

Step 1: Assessment		Step 2: If assessment is positive
Delirium triage screen [39]	98% sensitive for ruling out delirium. Time 10 s.	1. Physician administers CAM ICU. If positive, geriatrics consult ordered.
		3. Delirium precautions.
Four-Stage Balance Test [40]	Balance test that improves identification of older adults in the ED at risk for fall. Time 40 s	1. Fall precautions.
		2. Physical therapy consult.
		3. Case manager home safety evaluation
		4. Geriatrics consult.
Identifying Seniors at Risk [41]	6 questions on ability to care for self, memory, and medication. Time 90 s	1. Pharmacy consult if ≥ 5 medications.
		2. Case management consult if score ≥ 2.
		3. Geriatrics consult if score ≥ 2.

**Table 2:** Patient-centered outcomes chosen to evaluate the effectiveness of the protocol for multidisciplinary geriatric assessment in the emergency department

OARS: (Primary outcome) An assessment of activities of daily living (functional status) commonly used in ED studies. We will obtain 3 timepoints: at the ED visit (day 0) and at days 30 and 90. A change of ≥ 3 points or death between is a significant decline.	
HRQoL: The Patient-Reported Outcomes Measurement information System (PROMIS) is endorsed by the NIH and PCORI. We will use the Global Health v1.2 (10 questions). A 3-point change is clinically meaningful.	
New services: The number of new or increased outpatient services (e.g., home health therapies, referral for community interventions, referrals to hospice, equipment).	
New geriatric syndromes: Number of new diagnoses of delirium, impaired cognition, fall risk, or elder mistreatment.	
Geriatric clinic referral: Number of referrals to the falls prevention, polypharmacy, or geriatric clinic.	
Pharmacist recommendations: The number of medication-related problems/interactions or medication changes recommended by the pharmacy team.	
Positive geriatric interventions: ≥ 1 of new services, diagnoses, referrals, or pharmacist recommendations.	
ED revisits and hospitalizations: Any ED revisits or unscheduled hospitalizations within 90 days.	
Patient satisfaction: Thematic analysis of semi-structured interviews with participants.	

### Study design—implementation

This is a T3 translation study, where a treatment effect is studied in real clinical practice [43]. For the first aim, implementation, the proportion of older adult patients receiving geriatric screening will be evaluated pre- and post-intervention. The Consolidated Framework for Implementation Research: Care Transitions Framework (CFIR) was chosen to derive barriers and facilitators to implementation and map them to the external context, setting structure (organizational characteristics), provider roles and characteristics, and patient characteristics and factors (Table 3) [44]. CFIR has been used successfully in the ED setting to guide rapid cycle process improvement [45, 46].

**Table 3:** Study intervention characteristics as mapped to the Consolidated Framework for Implementation Research Transitions of Care Framework, adapted from Rojas Smith et al. [38]

Intervention characteristics	Vision of intervention: All older patients in the ED will be screened for cognitive problems, mobility issues, and home needs, and appropriate solutions will be found using a multidisciplinary approach.
	Target groups: Older adults in the Obs Unit and ED staff
	Intervention source: Administrative, led by ED physicians
	Evidence strength: Moderate. Positive results from similar programs at other institutions at reducing admissions and identifying unrecognized geriatric needs.
	Feasibility: Stakeholders in the implementation group and frontline feel this can be successfully carried out.
	Adaptability: Moderate to high. If we find screening tools insensitive or that this is impeding flow, the algorithm can be changed.
	Trialability: Intervention trialed on a small scale in the Obs Unit only, but now needs to be expanded to screening everywhere in the ED.
	Complexity: High, involves multiple screens, multiple consultants, and buy-in from multiple departments in the health system.
	User control: High. The intervention relies on staff action.
	Location of intervention activity: ED and Obs Unit.
	Task standardization: Screening tools and observation ordersets have been built into the electronic medical record.
External context	External pressures: Reducing readmissions and the payer mix in the area are pressures to implement this intervention. Additionally, Geriatric ED Accreditation is an external pressure to implement this program.
	External policies: Geriatric ED Accreditation and Accountable Healthcare Organization both advocate for addressing needs in the ED and coordinating with community care.
	Population needs: Demographics endorse this project. Aging population in the area is of high medical complexity, and access to care and specialists is often difficult.
	Community resources: Good availability of community resources for home health. Some difficulty with acute rehabilitation or skilled nursing facility placement from the ED.
Organizational characteristics	Structural: Hospital is mature, well respected, and well integrated into the community. Obs Unit is located within the ED and flexes beds with the ED. High ED boarding rates lead to a focus on reducing admissions. One barrier is that as a tertiary care facility, ED acuity is high and the focus on acuity may decrease the time needed for geriatric screening and management.
	Networks and teamwork: Communication between ED team, Obs team, and consultant teams is moderate. Formally communicate via health records, informal by phone calls. Communication between case management team and community resources is strong.
	Culture: Strong “flow culture” resistant to introduction of tasks that do not improve flow is a barrier to implementation. There is also some fatigue from frontline staff due to the constant march of quality improvement initiatives. Email is not a good way to disseminate information. Nursing culture does include “huddles” before every shift which is a good way to allow staff to question new projects and disseminate information.
	Implementation climate: Strong organizational push for better care of older adults. Hospital has NICHE certification and a modified ACE unit. Consultants are very willing to assist with the process. ED, RN, and hospital administration in favor of the project. Climate for trialing new processes and learning initiatives good.
	Tension for change: Low. Staff feel comfortable with the current status.
	Organizational mandate: Moderate (the organization is constantly mandating things).
	Accountability: Low, no tangible consequences for not following the intervention.
	Relative priority: Moderate. Most nurses will do the screening if asked, but it is not top priority for patient care.
	Readiness: Educational training not complete due to high nursing turnover. RN leadership may see this as a side project. The last QI project led by Dr. Southerland for ED nurses had 73% compliance among nurses for the survey and 81% compliance with a 1-h online training module. Therefore, the team has a track record of obtaining good compliance with training initiatives.
	Access to training: RNs are given protected time for training and have dedicated nurse educators (facilitator).
	Patient oriented: High patient orientation, project is focused on identifying and meeting patient needs. This is a strength in the eyes of the organization and staff (facilitator).
	Human factors
	IT accessibility: Geriatric screening tools and a flowsheet are built in but may require improvement to make it more noticeable and work with flow issues (barrier). Department has IT infrastructure for quality improvement projects (facilitator).
	Physical space/equipment: No new space or equipment needed
	Staff time: Large factor, however the assessments take last than 3 min.
Characteristics—provider roles	Nurses: Frontline team for this effort and will direct care. On the other hand, RNs feel overwhelmed and overburdened with patient care needs.
	Knowledge/beliefs: Will need knowledge refreshers as some did the training 2–3 years ago. As they spend the most time with the patient, they are most likely to recognize delirium or fall risk. There is some age bias, and some staff may be reluctant to go into an older adults’ room because of fear it will take longer to perform simple tasks.
	Skills/competency: Very skilled at screening questions, as this is a part of ED triage.
	Role: Initial screening (step 1) and informing the physician team.
	Self-efficacy: Very high. Emergency RNs take on a great amount of responsibility and are allowed to place triage orders.
	Physicians: Frontline team, the same physicians and advanced practice providers work in the ED and Obs Unit.
	Knowledge/beliefs: Focused on acute care mindset only. Will often ignore other issues that do not specifically cause problems in the ED setting (e.g., delirium, fall risk, polypharmacy). Education/knowledge level of the intervention is low.
	Skills/competency: Frequently use ordersets and order consultations.
	Role: (step 2) Determine appropriate multidisciplinary geriatric assessments to order.
	Self-efficacy: Very high.
	Consultant teams: Good culture of seeing patients quickly. Intervention should assist with their care and possibly speed their evaluations.
	Time: No dedicated staff time for this project other than nurse educator and PI (Dr. Southerland).
Characteristics—patients	Socioeconomic effects: Socioeconomic barriers may impede access to care and follow-up. Plan to control for this by using zip code-level socioeconomic status and type of health insurance as variables in the logistic regression.
	Cultural: We will only be able to recruit English-speaking patients into the pre-/post-cohorts, and so will not be able to assess the effect of cultural differences.
	Patient needs: May minimize symptoms. May not be receptive to interventions such as rehabilitation placement or home care options.
	Caregiver needs: May or may not have caregivers available. If available, case manager assesses for caregiver burden and assists with arranging care needs and medical equipment, if applicable.
	Other: Patients may be hesitant to speak out (elder abuse, cultural differences) or criticize their care during interviews.
Process of implementation	Lean Six Sigma
	Planning: Baseline focus groups to identify barriers. Workflow analysis to address the flow culture needs. Time for planning and lean meetings during the pre-implementation phase.
	Acquiring resources: No new resources or staff acquired.
	Process roles
	Process ownership: Dr. Southerland to be the ED physician lead, Erin Farrell (ED nurse manager), Peg Gulker, and Cole Briggs are nurse leaders.
	Organizational leaders: Chief Nursing Officer Beth Steinberg and Executive Director of the hospital, Dr. Susan Moffatt-Bruce.
	Opinion leaders/champions: Some opinion leaders already identified (charge RNs) but need to find staff RN champions for each unit.
	External change agents: Could consider involving local payer groups (Medicare groups)
	Integrators: Case managers may play a large role in furthering care by coordinating between the consultants and outpatient resources.
	Patients and caregivers: Study plans discussed with the ED Patient Advisory Board who were very positive and encouraging.
	Reflecting and evaluating: Quantitative feedback arranged in the form of a Geriatric ED dashboard. Currently monthly, but will change data reports to weekly during implementation.
Measures of implementation	Acceptability: Focus group interviews to determine barriers and examine shifts in culture. Before/after surveys of RNs and ED MDs to determine changes in knowledge and awareness.
	Appropriateness: Will be examined with the effectiveness data.
	Intervention cost: Not measuring. No new staff/costs for the ED, but potentially new healthcare costs for patients.
	Fidelity: Adherence to protocol based on chart audits by research staff. Study will also report how the initial protocol is adapted during implementation.
	Reach: Number of patients affected (15,000 older adult patients per year in the ED).
	Sustainability: Phased withdrawal of implementation procedures (chart audits, weekly meetings, etc.). Sustainability measures included including assessments at 1 and 2 years out.
	Evolvability: Number of rapid cycles needed to achieve > 80% screening rate. Changes to original protocol needed to meet changing ED environment or patient needs.
Outcomes	Patient centered: Multiple patient-centered outcomes include health-related quality of life, unmet needs identified and addressed, and functional status.
	Patient experience: Plan to assess via randomized semi-structured patient interviews in the post-cohort.
	Provider experience: The sustainability surveys address this and evaluate how easy and routine the intervention has become.
	Processes of care: Team will record ED admission rates and length of stay in the ED.
	Care coordination: How often are post-ED visit appointments made? How often is follow up completed in specialty clinics (falls, geriatrics, etc.)?
	Safety: Plan to record any patient complications or family concerns that arise during the screening/assessment process.
	Healthcare utilization: Readmissions, ED revisits, adherence to recommendations for physical therapy, occupational therapy, and geriatric referrals.
	Unintended consequences: Any increased pressures on workflow, difficulties with consultants, or overload of the Obs Unit staff.

*ED* emergency department, *Obs Unit* observation unit, *RN* registered nurse, *ACE* acute care of the elderly, *IT* information technology

The implementation method will be Lean Six Sigma, which is a commonly used tool for rapid cycle process improvement in healthcare settings. Lean Six Sigma is a QI approach that is validated in healthcare settings and used by our hospital [47]. Lean Six Sigma functions well for quality improvement in EDs [48–52], but there is minimal data on implementation strategies/methods in Obs Units [53, 54]. The Lean Six Sigma team will consist of frontline staff, nursing leaders, physician leaders, and members from PT, geriatrics, pharmacy, and case management teams.

The design and reporting of this study adhere to the Standards for Reporting Implementation Science (Table 4) [55]. The core component of the intervention is multidisciplinary geriatric assessment in the ED setting. The adaptable elements are the screening tools used, where and who does the screening, and where the geriatric assessments occur. For example, one study found that PT evaluations in the ED setting can be done without prolonging ED length of stay, but this service was only offered Monday through Friday, 7 am–4 pm [56]. The implementation team may discover that during regular business hours multidisciplinary assessments can be completed in a time frame that does not require observation placement. We would not consider this a “protocol violation” but an adaptation taking advantage of times when resources are high. Similarly, it may be discovered that there is a significant impediment to workflow with one of the chosen geriatric screening tools or that new data shows that a different tool has improved specificity. Any protocol adaptions and the reasons will be reported. Changes in knowledge and awareness will be assessed with before/after surveys.

**Table 4:** Standards for Reporting Implementation Studies study checklist and rationale for choosing measures to report, adapted from Pinnock et al. [55]

STARI Checklist item		Explanation	Study compliant?	
Title	1	Include identification as implementation study and methods used	Yes	
Abstract	2	Include description of implementation strategy to be tested, evidence-based intervention, and key implementation and health outcomes.	Yes	Framework: CFIR
				Strategy: Lean Six Sigma
Introduction	3	Include a description of the problem, challenge/deficiency that intervention aims to address.	Yes	
	4	Include the scientific background and rational for the implementation strategy and any pilot work.	Yes	
Aims and objectives	5	Differentiate between the implementation objectives and any intervention or healthcare outcome objectives.	Yes	Aim 1: Implementation
				Aim 2: Effectiveness
Methods: description	6	Include the design and key features of the evaluation and any changes to study protocol, with reasons.	Yes	Single site, pre-/post-cohort study
	7	Describe the context in which the intervention was implemented (social, economic, policy, healthcare, and organizational barriers and facilitators that influence implementation).	Yes	Plan to provide updated Table 3 if circumstances change significantly.
	8	Include the characteristics of the inner setting or target site (locations, personnel, resources, etc.).	Yes	
	9	Include a description of the implementation strategy.	Yes	Plan to report Lean Six Sigma elements, CFIR barriers targeted and concept map
	10	Describe any subgroups recruited for additional research tasks and or nested studies.	Not applicable	
Methods: evaluation	11	Include pre-specified primary outcome and any secondary outcomes of the implementation strategy and how they were assessed.	Yes	Goal of > 80% screening.
	12	Describe process evaluation objectives and outcomes related to the implementation strategy.	Yes	
	13	Describe methods of capturing resource use, cost, economic outcomes, and analysis.	No economic analysis.	
	14	Include rationale for sample sizes.	Yes	
	15	Describe methods of analysis and rationale for this choice.	Yes	
	16	Describe any a priori subgroup analyses.	Yes	
Results	17	Include proportion recruited and characteristics of the recipient population for the implementation strategy.	Yes	
	18	Report the primary and other outcome(s) of the implementation strategy.	Yes	
	19	Report the process data related to the implementation strategy (Lean Six Sigma), mapped to the mechanism by which the strategy is expected to work (improving capacity, opportunity, or motivation)	Yes	
	20	Include the resource use, costs, economic outcomes, and analysis for the implementation strategy.	No economic analysis.	
	21	Report the representativeness and outcomes of the subgroup recruited for research.	Yes	Will compare to all ED patients and all Obs Unit patients ages ≥ 65 years.
	22	Report the fidelity to implementation strategy as planned as well as any adaptations to suit context and preferences.	Yes	
	23	Include any contextual changes which may have affected outcomes.	Yes	
	24	Include all important harms or unintended effects in each group	Yes	Monitoring for unintended effects on ED and Obs Unit operational metrics.
Discussion	25	Summarize the findings, strengths and limitations, and compare with other studies.	Yes	
	26	Discuss the implications on policy and any potential impact with scaling the intervention.	Yes	
General	27	Include statements on regulatory approvals and trial/study registration.	Yes	

As part of the evaluation, we will analyze sustainability of the program. There are no sustainability instruments validated in the ED; we adapted the Measurement Instrument for Sustainability of Changed Work Practices (Sustainability survey) to evaluate culture and routine changes in this new setting [57]. This survey will be given to the 150 ED nurses at 12 and 24 months after full implementation, with attention to the sections on routinization and institutionalization.

### Measures and data analysis—implementation

Monthly geriatric patient visits as well as geriatric screening and consultations completed will be recorded and summarized descriptively (means, standard deviations, 95% confidence intervals; medians, interquartile ranges; proportions with 95% confidence intervals). For fidelity, we will look at the proportion that screened positive for consultation and received the appropriate consultation for their positive screening test. We will also perform fidelity checks during the post-cohort recruitment. We will note any external or internal events that may affect the process, such as new governmental or institutional mandates that may arise.

As a secondary analysis, we will examine the change in the proportion of geriatric screening using an interrupted time series analysis. We do not expect to see seasonal variation, but may see effects from confounders such as changes in total ED volume, nursing staff turnover (as evidenced by hours of float nurse pool coverage), patient volumes, and ED boarding rates [58].

### Power—implementation

This phase of the study is based on all geriatric patients seen in the ED. On average, 6660 patients are seen per month in the OSUMC ED with approximately 25% or 1800–2000 age ≥ 65 years. In October 2019, we had 7252 total patients, 1439 (19.8%) aged ≥ 65 years, of which only 20 were screened (1.3%). Assuming 1300 monthly geriatric visits, the two-sided 95% CI width for the monthly proportion for screening rates will range from 0.016 if the actual proportion is 1.5% to a maximum of 0.055 if the proportion is 50%. We will have balanced time periods of 24 months before and 24 afterwards. The study timeline is 5 years, which includes a year for implementation.

### Study design—effectiveness

The effectiveness study is a pre-/post-cohort analysis. We will evaluate *patient-centered outcomes—*functional status, health-related quality of life (HRQoL), and patient satisfaction (Table 2). Study personnel will monitor the Obs Unit electronic tracking board (7 am–11 pm M–F and select weekends) to identify and recruit 380 patients. A survey and chart review will be completed during the Obs Unit stay. Initial data elements collected include demographics, insurance status, zip code for socioeconomic status estimate, and the Charlson Comorbidity Index components and overall score [59]. Follow-up phone interviews will occur at days 30 ± 3 and 90 ± 5 post index visit. In simulations, survey completion required 8–10 min. Surveys include the Older Americans’ Resources and Services Activities of Daily Living questionnaire (OARS) and Patient-Reported Outcomes Measurement Information System (PROMIS) HRQoL, ensuring comparability to existing studies and ease of data dissemination [41, 60, 61]. Outcome scales will be measured during the ED stay, at 30 and 90 days. Primary outcome is the change in functional status based on OARS from 0 to 90 days. A planned subset analysis comparing the patients in the post-cohort who had the full screening and protocol to those in the pre-cohort who did not will be done.

To assess *patient satisfaction* with the protocol, a trained researcher will conduct in-person, semi-structured interviews with post-cohort patients. In addition to Likert-type questions, we will solicit brief descriptions of clinical exemplars of this intervention. Maximum variation/heterogeneous purposive sampling will provide a mix of genders, ages, and observation dispositions for the structured interviews [62]. Initial sampling will be sequential. After enrolling patient 30 into the post-cohort, the research team will begin qualitative semi-structured patient interviews. We will interview every 5th patient for 10 interviews. The team will then summarize the interviewee demographics and identify specific types of patients for the next 10 interviews to ensure that there is representative viewpoints of the oldest old (age 85+), the younger old (age 65–70), all genders, and admitted and discharged patients.

### Data analysis—effectiveness

The primary effectiveness outcome is the proportion of patients in the pre- and post-cohort with a significant (≥ 3 point) decline in functional status (OARS) from day 0 to day 90. This corresponds to a complete loss of one activity of daily living or a decrease in several. We will compare proportions with decline in the pre- and post-group using a chi-square test (primary analysis). Additionally, logistic regression modeling will be used to compare the functional decline between the groups univariately and while controlling for initial ED HRQoL, demographic factors (age, race, average socioeconomic status from zip code census tract), Charlson Comorbidity Index score [43], home health services prior to the ED visit, and any other significant factors varying between the two cohorts. The total number of covariates will not exceed 12, given we estimate 122 subjects with functional decline. A similar secondary analysis will be done using the 30-day timepoint data.

For secondary outcomes, proportions of dichotomous variables will be compared between pre- and post-intervention cohorts using chi-square tests. Regression models will be used for exploring additional secondary outcomes between the two groups. The model used will be dictated by the outcome of interest such as logistic regression for dichotomous outcomes and Poisson or negative binomial regression for count data.

Qualitative analyses will be conducted from the patient interviews. Patient interviews will be transcribed verbatim and entered into Atlas.ti (Scientific Software Development GmbH). We will conduct manifest content analysis of the descriptions using phrases and sentences as our unit of analysis [63, 64]. We will categorize the exemplars and analyze for appropriateness and perceived outcomes based on the level of detail provided. Analysis will include open and axial coding procedures using techniques of constant comparison and questioning within and across cases [65, 66]. The coding schema will be created with consensus on coding definitions and grouping codes into code families/categories. Dual coding with negotiated consensus will be performed on 20% of the data to add rigor to the analysis. Themes will be reported per the consolidated criteria for reporting qualitative research guidelines [55, 67].

### Power—effectiveness

We conservatively estimate that 40% of the pre-cohort and 30% of the post-cohort will experience functional decline [19, 68–70]. We will over-sample the post-implementation cohort due to the expected heterogeneity (screen negative/positive, with/without full intervention). With 137 subjects in the pre-intervention cohort and 206 in the post-intervention cohort, we will have 80% power to detect a difference of this magnitude, based on a chi-square test and an alpha of 0.05. To account for an estimated 10% loss to follow-up, 380 subjects (150 in the pre-cohort and 230 in the post) will be recruited.

## Discussion and dissemination

This single-center, hybrid implementation/effectiveness study is the first study of multidisciplinary geriatric assessment in the ED setting which is designed to be reproducible and sustainable without external funding or increasing ED length of stay. By incorporating consultants to assist with multidisciplinary assessment, the program also does not require new staff. These were all considerations by the interdisciplinary geriatric ED team which developed this protocol. The protocol has been piloted and now requires a full implementation and evaluation. By assessing implementation factors and evaluating sustainability, the study will provide information on the implementation of protocols and screening tools in an acute care setting. This information will also be used to plan a subsequent dissemination study of the protocol if it proves to be effective for reducing functional decline and poor outcomes after an ED visit.

Publishing the full study protocol is meant to encourage discussion about implementation in acute care and short stay settings. Lean Six Sigma was chosen as the implementation strategy because it provides a framework for rapid cycle process improvement, as well as specific strategies for handling complicated systems with a variety of inputs. Lean Six Sigma is also oriented towards improving flow, which is critical to the time-sensitive ED setting. It has a focus on including frontline staff in all aspects of quality improvement, standardizing and simplifying processes and protocols, and optimizing the value to customers. In this case, both values to the hospital and to the patient will be assessed by including both hospital operational metrics as well as patient-oriented outcomes. CFIR was chosen because the transition of care framework considers all the moving parts and different people involved in emergency care, including population and community needs, patient-centeredness, different levels in the health system, and appropriate outcome measures for this type of study.

We specifically chose not to include an economic analysis as part of the study outcome (Table 4). The goal of this program is to improve the transition of care from the ED to home, allowing older adults to live safely in the community. Therefore, this program will initially increase healthcare costs. An economic analysis of healthcare cost savings should be done over the course of ensuing years, not 90 days. In essence, one could consider this study the pilot to determine if the ED effectively identifies needs and connects the older adult patient to resources, and further studies can assess for downstream healthcare cost savings if the program is successful.

This study hopes to develop a method of incorporating holistic, geriatric care into the ED setting in a sustainable fashion. The authors invite any input on the design of this and future studies that could arise from this data.

## Data Availability

Final study data will be made available by request.

## Abbreviations

CFIR: Consolidated Framework for Implementation Research: Care Transitions Framework
ED: Emergency department
EHR: Electronic health record(s)
HRQoL: Health-related quality of life
OARS: Older Americans’ Resources and Services Activities of Daily Living questionnaire
Obs Unit: Emergency department observation unit
PROMIS: Patient-Reported Outcomes Measurement Information System
PT: Physical therapist or physical therapy
